# Multidrug Resistance and Virulence Factors of *Escherichia coli* Harboring Plasmid-Mediated Colistin Resistance: *mcr-1* and *mcr-3* Genes in Contracted Pig Farms in Thailand

**DOI:** 10.3389/fvets.2020.582899

**Published:** 2020-11-10

**Authors:** Nwai Oo Khine, Kittitat Lugsomya, Benjarong Kaewgun, Lertrob Honhanrob, Panupong Pairojrit, Suthipat Jermprasert, Nuvee Prapasarakul

**Affiliations:** ^1^Department of Veterinary Microbiology, Faculty of Veterinary Sciences, Chulalongkorn University, Bangkok, Thailand; ^2^The International Graduate Course of Veterinary Science and Technology, Chulalongkorn University, Bangkok, Thailand; ^3^Department of Infectious Diseases and Public Health, Jockey Club College of Veterinary Medicine and Life Sciences, City University of Hong Kong, Kowloon, China; ^4^Diagnosis and Monitoring of Animal Pathogen Research Unit (DMAP), Bangkok, Thailand

**Keywords:** colistin resistance, *Escherichia coli*, *mcr* genes, pigs, virulent factor

## Abstract

The presence of the plasmid-mediated colistin resistance encoding *mcr* gene family in the *Enterobacteriaceae* is one of the crucial global concerns. The use of colistin in livestock rearing is believed to be the cause of *mcr* gene spreading and is of impact to public health. The objective of this research was to detect the frequency and virulent genes of *mcr-*positive *Escherichia coli* (MCRPE) in fecal samples from healthy pigs in a contract farming system across Thailand. A total of 696 pooled samples were derived from 80 farms, located in 49 provinces across six regions of Thailand. The colistin-resistant *E. coli* were identified by MALDI-TOF mass spectrometry and antimicrobial susceptibility testing by broth microdilution. The antibiogram was determined using an automated susceptibility machine, and the genetic characteristics were investigated for *mcr-1–5* genes, phylogenetic group, replicon types, and virulent genes. In total, 31 of 696 samples were positive, with *E. coli* containing *mcr-1* or combination of *mcr-1* and *mcr-3* with incidence of 4.45 and 0.43%. Phylogenetic groups A and B1 and the IncF and IncFIB replicon types were predominantly found in the MCRPE located in the central area, with multidrug-resistant traits against 3–14 types of antimicrobials. Additionally, 19 of 31 isolates identified as enterotoxigenic *E. coli* were with the *stap* and *stb* (enterotoxin-encoding genes). In conclusion, a low carriage rate of *mcr-*positive *E. coli* was detected in the large-scale farming of healthy pigs. The association between multidrug-resistant MCRPE and their pathogenic potential should be of concern.

## Introduction

Antimicrobial resistance (AMR) is an emerging concern for both human and animal sectors of the world. The inappropriate use of antimicrobials in clinical settings and, most importantly, in livestock farming imposes social and economic burdens on society (1). The diminishing number of active (effective) antimicrobial agents to treat sick farm animals is accompanied by the downfall in food production and the likelihood of exposure of farmers to resistant bacteria. *Escherichia coli*, a commensal microbe, can accumulate resistance genes. It is widely used as a representative example for monitoring resistance genes, especially for horizontal gene transfer (2). Therefore, the assessment of mobile genetic elements from commensal *E. coli* could highlight the AMR transmission between hosts (3).

Colistin is a cationic antibiotic that has long been regarded as a last resort antibiotic for *Enterobacteriaceae* infections. However, the widespread use of colistin in animal production acts as a selective pressure for the spread of plasmid-mediated colistin resistance genes, which are in the *mcr* family. The first discovery of plasmid-mediated colistin resistance (*mcr-1* gene) in *E. coli* from China raised an enormous attention globally and was followed by the subsequent discovery of other *mcr* resistance genes, including *mcr-2, mcr-3, mcr-4*, and *mcr-5*, in different geographical areas (4). Recently, another four colistin resistance genes (*mcr-6, mcr-7, mcr-8*, and *mcr-9*) were identified mainly from members in the *Enterobacteriaceae* family (5–8). Among them, *mcr-1* is the most frequently detected in farmed animals and from *Enterobacteriaceae* infections in humans (9). These reports raised awareness upon colistin usage, especially in livestock animals.

In Thailand, over 80% of pig farming systems are contract farming between the primary producers and the agribusiness companies, for the latter to procure a certain pre-agreed quality and quantity of products at an economical price and is lesser from the primary producers. Antimicrobials including colistin are feed additives or prophylactic agents, including colistin, against bacterial infections in pig farms under veterinary prescription (10). Although there have been a few reports regarding a high prevalence (60–90%) of multidrug-resistant (MDR) *E. coli* in pigs in Thailand, the antimicrobials used on the farms have not always been clearly defined (11). Since colistin resistance is the crucial epidemiological data of public health concern, monitoring the prevalence of colistin-resistant *E. coli* and their characteristics is of high priority. The objective of this study was to characterize the antibiogram and virulent traits of *mcr*-positive *E. coli* (MCRPE) from the fecal samples of healthy pigs derived from the contract farming system across Thailand.

## Materials and Methods

### Study Area and Animal Selection

Samples were collected from 80 farms, in 49 provinces across six regions of Thailand, comprised of 15, 5, 12, 7, 4, and 6 provinces from central, northern, northeastern, eastern, western, and southern Thailand, respectively. Farms were selected based on the available management data, including the antimicrobial usage, housing, vaccination, feed type, and production cycle. However, all historical data was allowed as inclusion criteria for farm selection only but not allowed to be included in the analysis. A total of 696 pooled fecal samples (5–10 samples per farm) were collected from individual 18- to 20-weeks-old fattening pigs with a normal clinical appearance and no recent history of enteric disease or therapeutic antimicrobial treatment.

### Sample Collection and Bacterial Identification

At least 5 g of feces per pig was collected into a sterile container and kept at 4°C until processed. Then, the fecal samples were homogenized and mixed to get pooled fecal samples with a total mass of 25 g. Then, 5 g of well-mixed feces was collected and diluted 10-fold using sterile 0.85% (w/v) NaCl. Dilutions of 10^−7^-10^−8^ were spread on eosin methylene blue agar (Oxoid, UK) plates containing 2 μg/ml colistin sulfate (Sigma-Aldrich, USA) to select for the presumptive colistin-resistant *E. coli*. The biohazard execution control was approved by the Institutional Biosafety Committee of the Faculty of Veterinary Science, Chulalongkorn University (IBC 1731021). One representative colony with typical *E. coli* morphology was picked and subcultured to get pure culture. The *E. coli* species was confirmed using matrix-assisted laser desorption ionization combined with time-of-flight analysis (MALDI Biotyper, Bruker, USA). The principle behind MALDI-TOF is based on mass spectrometry and “soft” ionization technique. Depending on the time of flight of each pathogen, the characteristic spectrum will be analyzed and displayed *via* the inbuilt software. Briefly, the bacterial colony sample was smeared as a thin film directly on a target plate and then coated with 1 μl polymeric matrix (a saturated solution of α-cyano-4-hydroxycinnamic acid in 50% acetonitrile and 2.5% trifluoroacetic acid) and air-dried at room temperature. This matrix could penetrate the cell wall of microorganisms and able to extract proteins. The target plate was placed into the mass spectrometer and irradiated by a laser. Afterwards, the molecules vaporized and ionized at the same time into the vacuum and transported to the detection device. Lastly, the computerized database results compared with the reference library database were generated with interpretations (12).

### Antimicrobial Susceptibility Determination and *mcr* Gene Detection

For colistin, the broth microdilution procedure was performed according to the Clinical and Laboratory Standards Institute (CLSI) recommendation (13). The plasmid-mediated colistin resistance genes (*mcr-1–5*) were detected by multiplex (m)PCR using GoTaq® Green Master Mix (Promega, USA) and the previously reported primers and PCR conditions (14). The *E. coli* strain CUP13 (15), which is positive for *mcr-1* and *mcr-3* (confirmed by Sanger sequencing), and ATCC25922 were used as positive and negative controls, respectively. Briefly, the thermocycling conditions were performed at 94°C for 15 min, followed by 25 cycles of 94°C for 30 s, 58°C for 90 s, and 72°C for 1 min, and then followed by 72°C for 10 min.

The minimal inhibitory concentration (MIC) of antimicrobial agents against the *E. coli* isolates was determined using the AST-GN 38 test kit in a Vitek2 compact automated susceptibility level detection apparatus (BioMérieux, France). The antimicrobial groups selected were synchronized with veterinary guidelines (16). Justification of the antibiotics chosen is for AMR monitoring and for the purpose of public health awareness such as the second generation of cephalosporin, aminoglycoside, fluoroquinolone, and carbapenem. *E. coli* ATCC 25922, *Pseudomonas aeruginosa* ATCC 27853, and *Staphylococcus aureus* ATCC 25913 were used as the control strains. The antimicrobials selected were amikacin (AK), amoxicillin (AMX), amoxicillin/clavulanic acid (AMC), ampicillin (AMP), cefalexin (CEX), cefpodoxime (CPD), cefovecin (INN), ceftiofur (XNL), chloramphenicol (C), enrofloxacin (ENR), gentamicin (GEN), imipenem (IMP), marbofloxacin (MBR), nitrofurantoin (NIT), piperacillin (PIP), tetracycline (TET), tobramycin (TOB), and trimethoprim/sulfamethoxazole (SXT). The MIC interpretations will be reported according to Food and Drug Administration (FDA) (17), CLSI (13), and EUCAST values (18). The isolates that presented an extended-spectrum beta-lactamase (ESBL) phenotype were confirmed with a double disc synergy test and phenotypic disc confirmatory test as previously reported (19).

### Phylogenetic Grouping

The MCRPE isolates were determined using an approved mPCR identification of their phylogenetic groups and subgroups (A, B1, B2, C, D, E, and F) as reported (20). Each reaction was performed in a 25-μl mixture containing 12.5 μl of GoTaq® Green Master Mix (supplied with *Taq* polymerase), 20 pmol of each primer, and 200 ng of genomic DNA. The *E. coli* ATCC 25922 and *E. fergusonii* CUVET427 (21) strains were used as the controls.

### Plasmid Replicon Typing

The *Enterobacteriaceae* plasmid replicons IncF (IncFIA, IncFIB, IncFIC, and IncFrep), IncI1-Ig, IncN, IncP, IncW, IncHI1, IncHI2, IncL/M, IncT, IncA/C, IncK, IncB/O, IncX, and IncY were detected using five mPCR and three simplex PCR tests. The primers, PCR conditions, and thermal cycles were applied as previously reported (22). Briefly, PCR amplifications, except the F-simplex, were thermal cycled at 94°C for 5 min, followed by 30 cycles at 94°C for 1 min, 60°C for 30 s, and 72°C for 1 min, and then followed by 72°C for 5 min. The F-simplex PCR was performed with the same amplification program except at an annealing temperature of 52°C. Positive control samples were provided and used as reported (21).

### Detection of Virulence Genes

The sets of mPCR and simplex PCRs were performed as previously reported (23), with the positive control strains taken from the previously sequenced enterotoxigenic *E. coli* (ETEC) and enterohemorrhagic *E. coli* (EHEC) strains (24). Primers specific for the *StaP* (heat-stable toxin a subdivide p), *Stb* (heat-stable toxin b), *Stx2e* (Shiga toxin), *K88* (Fimbriae), *F4* (Fimbriae), and *Ltb* (heat-labile enterotoxin b subunit) genes were used. The PCR assays were prepared with GoTaq® Green Master Mix (Promega, USA) and thermocycled at 94°C for 10 min, followed by 30 cycles of 94°C for 30 s, 55°C for 45 s, and 72°C for 1.5 min increasing by 3 s each cycle, and then followed by 72°C for 10 min.

### Data Analysis

The colistin resistance rates are presented as percentages divided by region and province in comparison of the rate with and without the *mcr* genes, and the antimicrobial resistance profiles are reported as the antibiogram patterns of *mcr-*positive *E. coli*. The patterns of virulence gene profiles among MCRPE isolates are presented in percentages. To define MDR and pathogenic traits among the colistin-resistant *E. coli*, the relation between AMR phenotypes and pathotype characteristics was analyzed using Fischer's exact test (*p* ≤ 0.05).

## Results

### Distribution of Colistin-Resistant *E. coli* Containing *mcr* Genes

**Figure 1:**
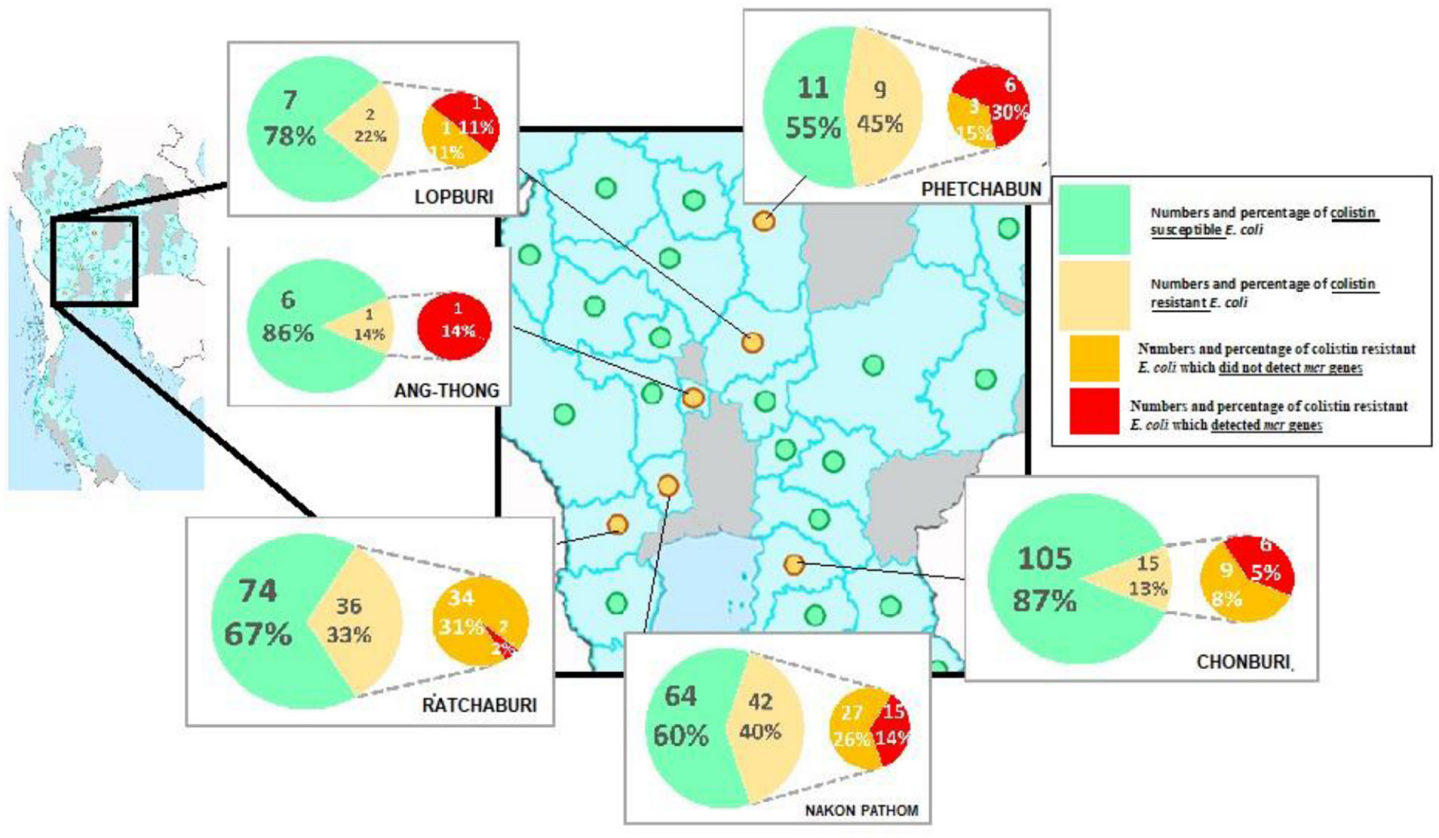
Geographical distribution of either colistin resistant or susceptible *E. coli* from the surveyed contracted pig farms in Thailand.

A total of 105 colistin-resistant *E. coli* from the 696 samples were isolated using the eosin methylene blue (EMB) media. From the broth microdilution method, the MCRPE isolates had MIC values of 4 (*n* = 17) or 8 (*n* = 14) μg/ml. From the PCR detection, the *mcr-1* gene was found in 31 of these 105 colistin-resistant *E. coli* isolates, and among them, three isolates were found to also express *mcr-3*. The distributions of colistin-resistant *E. coli* were from central (5.4%) (Phetchabun, Nakhon Pathom, Ang-Thong, and Lopburi), western (0.4%) (Ratchaburi), and eastern (1.4%) (Chonburi) Thailand. The geographical distributions of *E. coli* with or without *mcr* genes are shown in Figure 1.

### Antimicrobial Susceptibility Testing

**Figure 2:**
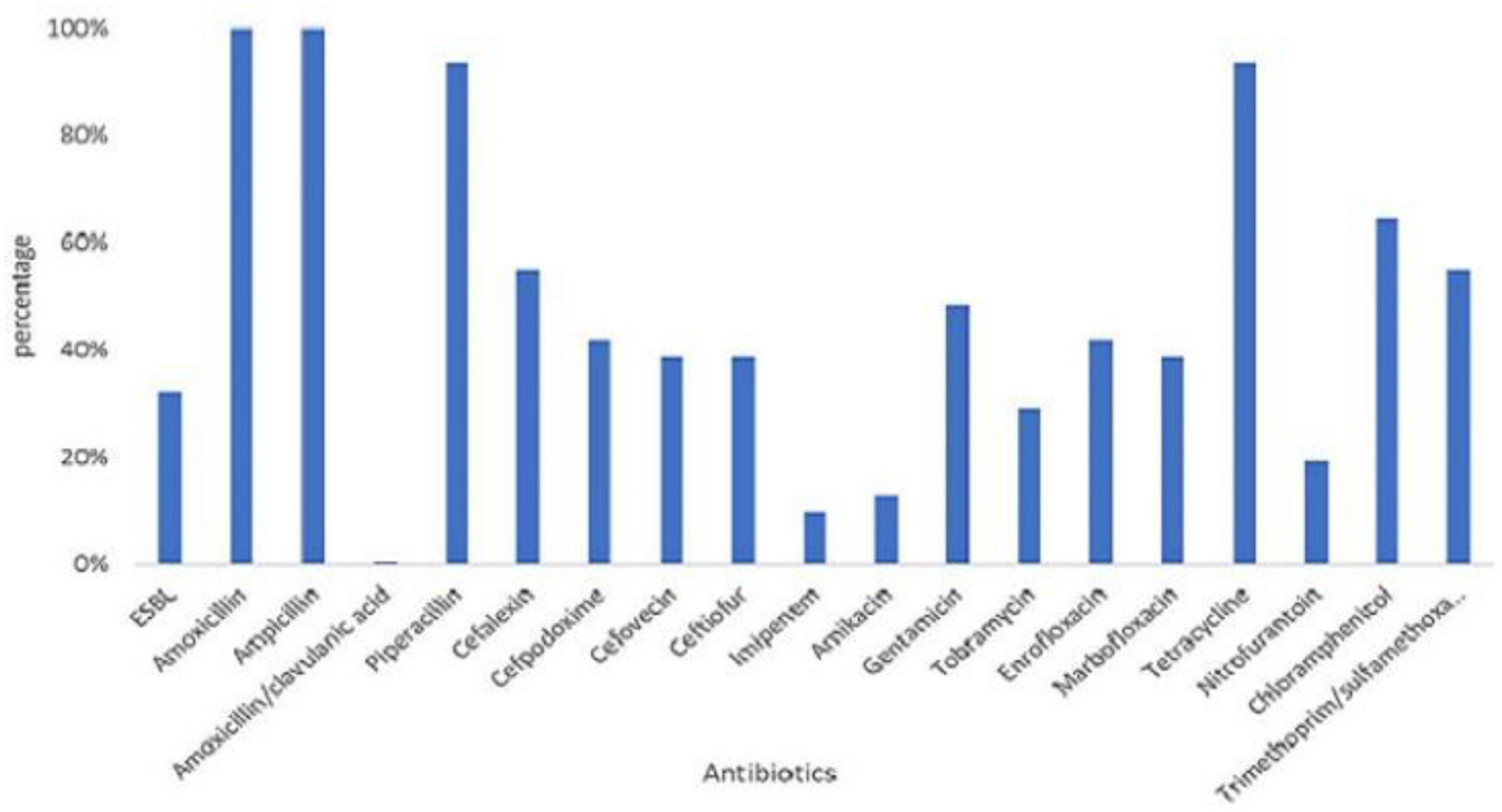
Distribution of resistant rates against 18 antimicrobials and presence of extended- spectrum beta-lactamase (ESBL) characteristic among 105 colistin-resistant *E. coli* isolated from contracted pig farms in Thailand.

All 31 MCRPE were multidrug resistant (Figure 2), with all being resistant to AMX, AMP, PIP, and TET, and over 50% were resistant to CEX, INN, XNL, GEN, ENR, C, and the SXT combination. No pan-drug resistance was detected among the MCRPE isolates. ESBL was found in 32.3% (10/31) *mcr-1* positive isolates. A total of 26 antibiogram patterns were recorded for 31 MCRPE isolates. Forty-eight percent (15/31) of these isolates were MDR with resistance to six antimicrobial groups (Table 1).

**Table 1:** Antibiograms of the 31 MCRPE isolates distributing in 26 pattern types.

**Pattern**	**Profile**	**Number of resistant ABOs**	**Isolate(s)**
A	AMX–AMP–PIP–CEX–CPD–INN–XNL–GEN–ENR–MBR–TET–C–NIT–SXT^*^	14	1
B	AMX–AMP–PIP–CEX–CPD–INN–XNL–IMP–AK–GEN–ENR–TET–C–SXT^*^	14	1
C	AMX–AMP–PIP–CEX–CPD–INN–XNL–GEN–TOB–ENR–MBR–TET–C^*^	13	3
D	AMX–AMP–PIP–CEX–GEN–TOB–ENR–MBR–TET–NIT–C–SXT	12	1
E	AMX–AMP–PIP–CEX–CPD–INN–XNL–IMP–AK–C–SXT^*^	11	2
F	AMX–AMP–PIP–CEX–CPD–INN–XNL–GEN–TOB–TET–NIT^*^	11	1
G	AMX–AMP–PIP–CEX–CPD–INN–XNL–TET–C–NIT–SXT^*^	11	1
H	AMX–AMP–PIP–CEX–CPD–INN–XNL–GEN–TOB–TET–C^*^	11	1
I	AMX–AMP–PIP–GEN–TOB–ENR–MBR–TET–C–SXT	10	2
J	AMX–AMP–PIP–CEX–CPD–INN–XNL–TET–C–SXT	10	1
K	AMX–AMP–PIP–CEX–CPD–INN–XNL–GEN–TET	9	1
L	AMX–AMP–PIP–CEX–ENR–MBR–TET–C–SXT	9	1
M	AMX–AMP–PIP–ENR–MBR–TET–NIT–C–SXT	9	1
N	AMX–AMP–PIP–CEX–ENR–MBR–TET–SXT	8	1
O	AMX–AMP–PIP–ENR–MBR–TET–C–SXT	8	1
P	AMX–AMP–PIP–GEN–ENR–MBR–TET–SXT	8	1
Q	AMX–AMP–PIP–GEN–TOB–TET–C–SXT	8	1
R	AMX–AMP–PIP–CEX–CPD–AK–TET	7	1
S	AMX–AMP–PIP–CEX–TET–C–SXT	7	1
T	AMX–AMP–PIP–TET–C–SXT	6	1
U	AMX–AMP–PIP–GEN–TET–NIT	6	1
V	AMX–AMP–PIP–TET–NIT	5	1
W	AMX–AMP–PIP–TET–C	5	2
X	AMX–AMP–GEN–TET	4	1
Y	AMX–AMP–PIP–TET	4	1
Z	AMX–AMP–TET	3	1

*AMC, amoxicillin–clavulanic acid; AMP, ampicillin; AMX, amoxicillin; C, chloramphenicol; CEX, cephalexin; CPD, cefpodoxime; ENR, enrofloxacin; GEN, gentamicin; MBR, marbofloxacin; PIP, piperacillin; SXT, trimethoprim/sulfamethoxazole; INN, cefovecin; AK, amikacin; IMP, imipenem; TET, tetracycline; XNL, ceftiofur; TOB, tobramycin; NIT, nitrofurantoin*.

* *ESBL*.

### Phylogenetic Grouping

**Figure 3:**
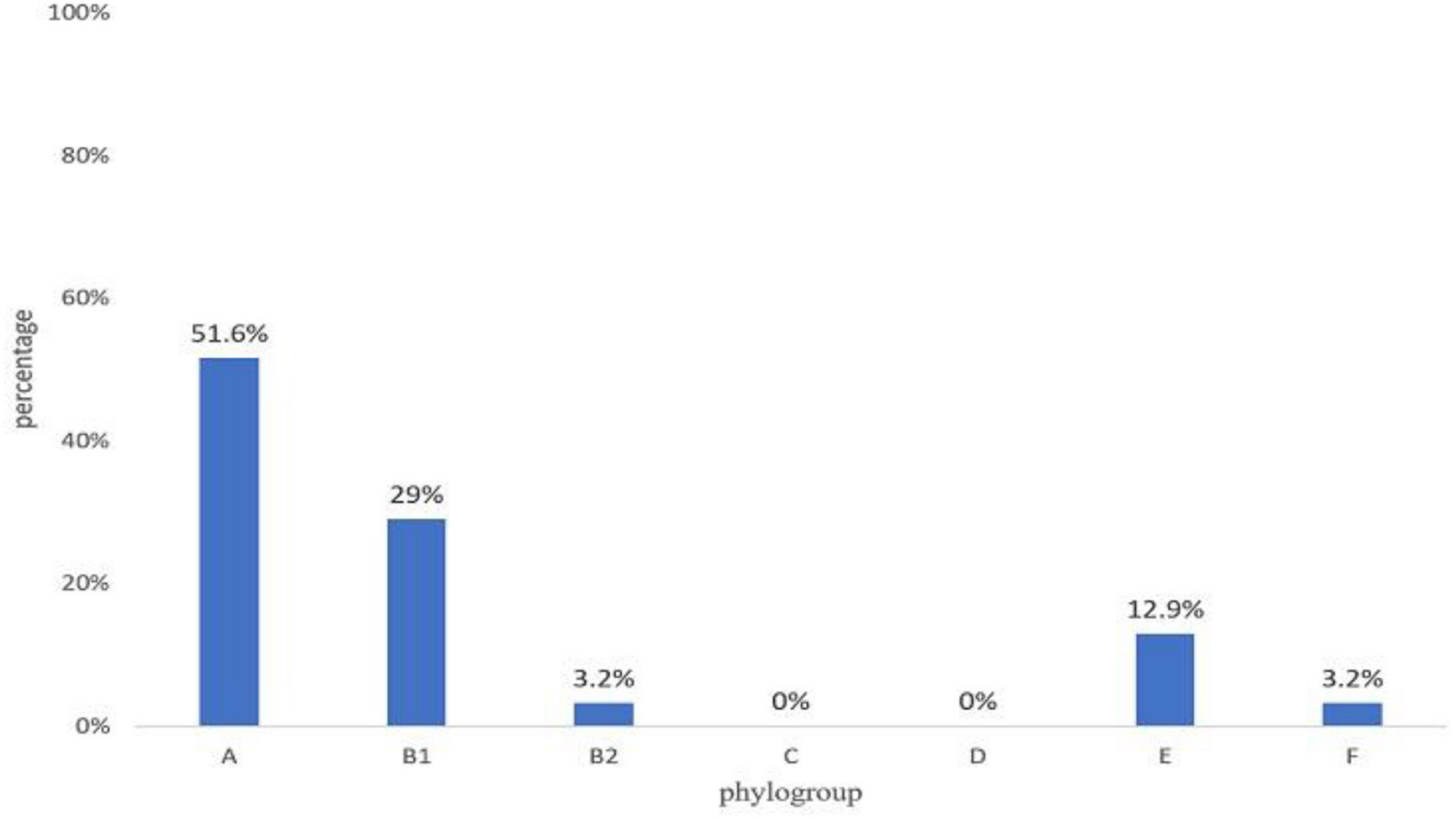
The phylogroups detected among 31 MCRPE isolates in contracted pig farms in Thailand.

Most isolates were from phylogenetic group A (51.6%), followed by group B1 (29%) and groups E (12.9%), B2 (3.2%), and F (3.2%) (Figure 3).

### Plasmid Replicon Typing

**Figure 4:**
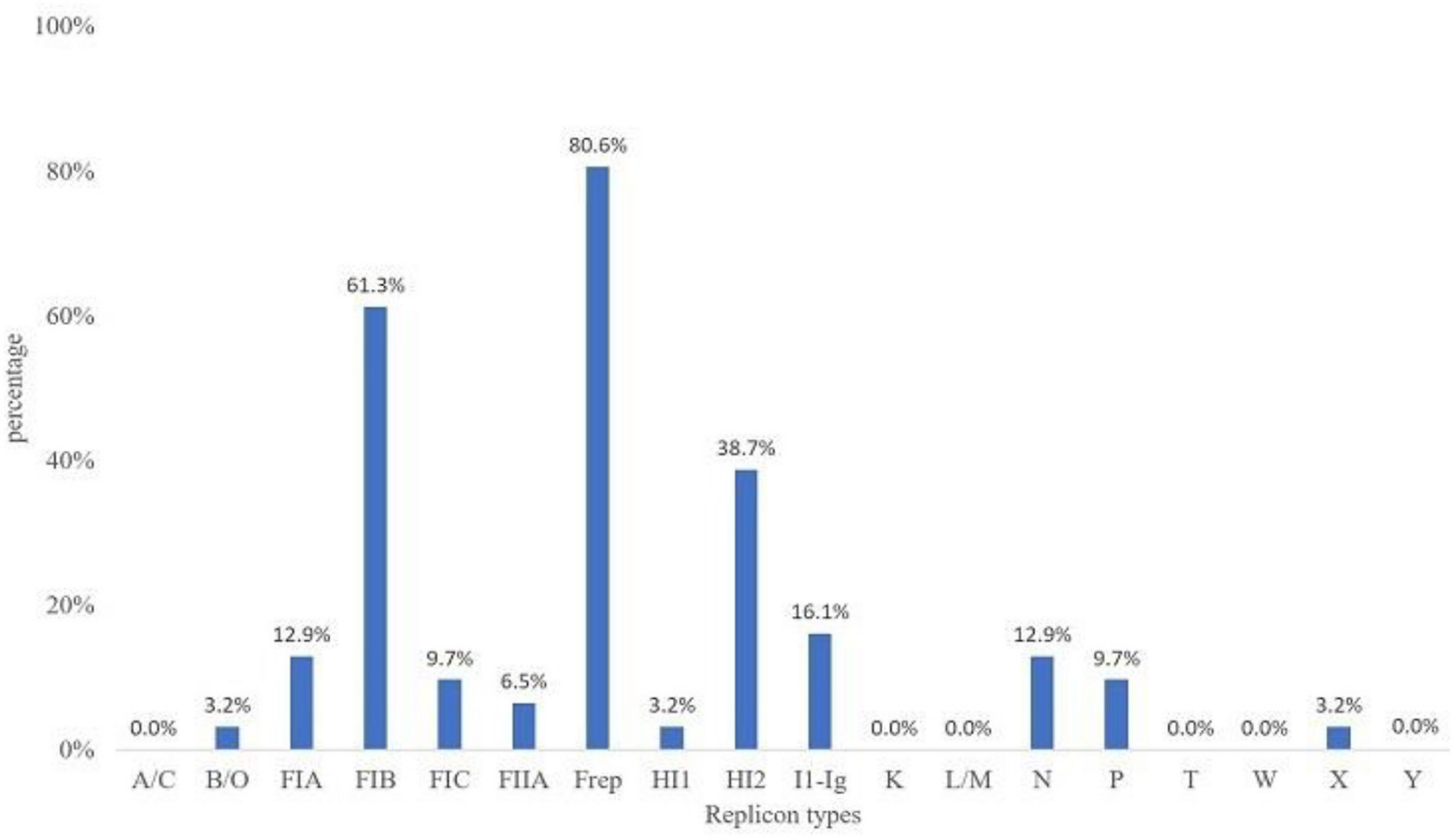
Plasmid replicon types detected among 31 MCRPE isolates in contracted pig farms in Thailand.

The predominantly found plasmid replicons were of the IncF and IncFIB replicon types at 80.6 and 61.3%, respectively. Plasmid replicon types L/M, W, Y, A/C, T, and K were not detected in this study (Figure 4). The other replicon types were found at low prevalence rates among the MCRPE isolates, with IncX, IncB/O, and IncHI1 being present at the lowest percentages (3.2%).

### Characterization of the Virulent Factors

The virulent genes representing ETEC or EHEC were found in 18 out of 31 (58.1%) MCRPE isolates (Table 2). The ETEC strains possessed the *StaP* and *Stb* enterotoxin-encoding genes as the most frequent pathotype, and one strain (from Phetchabun province) showed a hybrid ETEC–EHEC genotype.

**Table 2:** Presence of virulent profiles including toxin and antigenicity of the 31 MCRPEs.

**Virulence genes**	**ESBL (%)**	**Pathotype(s)**	**Number**	**%**
*StaP*–*Stb*–*Stx2e*	0	ETEC, EHEC	1	3.2
*StaP*–*Stb*–*K88*	3.2	ETEC	1	3.2
*StaP*–*Stb*	16.1	ETEC	13	41.9
*StaP*	3.2	ETEC	3	9.7
*Ltb*	0	ETEC	1	3.2
Negative	9.7	Non-pathogenic	12	38.7

*ETEC, enterotoxigenic E. coli; EHEC, enterohemorrhagic E. coli; Stap, heat-stable toxin a subdivide p; Stb, heat-stable toxin b; Stx2e, Shiga toxin; K88, Fimbriae, F4; Ltb, heat-labile enterotoxin, b subunit*.

### Relation Analysis Between Antimicrobial Susceptibility and Pathogenicity

The association between antimicrobial susceptibility and pathogenicity of the 31 MCRPE isolates was analyzed by Fischer's exact test (Table 3). No association between pathogenicity and resistance to the six antibiotic groups was found (fluoroquinolones, sulfonamides, tetracyclines, nitrofurazones, phenicols, and aminoglycosides) (*p* = 0.28, 1.00, 1.00, 1.00, 1.00, and 0.15, respectively).

**Table 3:** Relation analysis between MCRPE resistance to the other six antimicrobial groups and their pathogenicity.

**Antimicrobial group**	**Pathogenicity**	**Resistant**	**Susceptible**	***p*-value**
Aminoglycosides	Non-pathogenic	9	4	0.15
	Pathogenic	7	12	
Fluoroquinolones	Non-pathogenic	7	6	0.28
	Pathogenic	6	13	
Tetracyclines	Non-pathogenic	11	2	1.00
	Pathogenic	17	2	
Nitrofurazones	Non-pathogenic	3	10	1.00
	Pathogenic	4	15	
Phenicols	Non-pathogenic	9	4	1.00
	Pathogenic	12	7	
Sulfonamides	Non-pathogenic	7	6	1.00
	Pathogenic	11	8	

*Aminoglycosides: amikacin, gentamicin, and tobramycin; fluoroquinolones: enrofloxacin and marbofloxacin; tetracyclines: tetracycline; nitrofurazones: nitrofurantoin; phenicols: chloramphenicol; sulfonamides: trimetroprim/sulfamethoxazole; pathogenic: ETEC, ETEC–EHEC; non-pathogenic: negative for virulence genes*.

## Discussion

This national-scale study of contract-farmed pigs in Thailand confirmed the existence of colistin-resistant *E. coli* containing *mcr* genes and that they showed diversity in their phylogenetic group, replicon type, antibiogram, ESBL trait, and pathogenic potential. All recruited contracted pig farms had strict historical data and management records that can be traced back as an essential inclusion criteria. The sample collection criteria were set up and executed by the farm workers under the authority of veterinarians. In this study, MALDI-TOF MS was used for the identification and confirmation of bacteria strains. This technique has emerged as a powerful technique for the identification of microorganisms with an overall 95% accuracy at the species level. The main advantage of MADLI-TOF is being able to identify bacterial species directly from the culture plates as fast as 1 to 15 min in a few simple steps (12).

According to mPCR, our results indicated the lower resistance rate of *mcr-1* (4.4% or 31/696) when compared with a previous report from healthy pigs in China (21%) (20). This study covered all parts of Thailand where high-intensity pig farming is done. Unfortunately, all the historical data could not be analyzed due to the company's policy. However, the positive areas were distributed in the western, central, and eastern parts within a radius of about 300 km. The distributions of colistin-resistant *E. coli* were higher (15–30%) in Nakhon Pathom, Ratchaburi, Chonburi, Lopburi, and Phetchabun provinces. These provinces reported to have a huge number of pig farms and total number of pigs. Colistin was legally use in pig feeds for prophylactic purposes in Thailand until March 2018. The high percentage of MCRPE isolates in certain provinces might come from prolonged cumulative selective pressure from their history of colistin usage in pig feeds. To the best of our knowledge, this is the first report of *mcr-1* gene in *E. coli* isolates from pigs in Thailand. Interestingly, three of the *mcr-1*-positive isolates also co-expressed *mcr-3*. These results could highlight the awareness of the distribution of *mcr* genes and for the national policy of livestock immigration. The *mcr-1* genes have been widely shown to be distributed in Asia, Europe, Africa, and America and primarily due to the consequence of long-term colistin application in animals (25). The *mcr-3* gene was first reported in China in 2017 (26) and the prevalence and spread of the *mcr-3* gene in Thailand should be carefully monitored from now on.

According to phylogenetic grouping, the majority of the isolates in our study were in phylogroups A or B1, predominantly related with commensal strains (27). On the other hand, for the virulent *E. coli* groups, phylogroup D was not detected in the current study and there was a low frequency of phylogroup B2. Several studies have reported that phylogroups B2 and D were associated with intestinal and extraintestinal pathogenic *E. coli* as well as MDR strains (28, 29). Nonetheless, even commensal *E. coli* from various phylogroups have been reported to harbor pathogenicity islands that can serve as integration sites for virulence and/or AMR determinants (30) and so may facilitate in converting commensal strains to pathogens.

With respect to plasmid replicon typing, the IncFIB and F plasmids were the most commonly found replicon types in this study. They are narrow host-range-type plasmids, which have been reported in worldwide members of the *Enterobacteriaceae* family, associated with various antimicrobial-resistant genes (31). The *mcr-1* and *mcr-3* genes were previously described on the IncI, IncHI2, and IncX4 plasmids (32). A variety of replicon types were found in the MCRPE isolates in this study, which suggest that the *mcr* genes can locate and/or transfer to different plasmid types. This is in accordance with a previous report that the *mcr-1* genes and ESBL could be co-transferred by more than one type of conjugative plasmid, which might alleviate their effective dissemination among bacteria (33).

The antibiogram profiles characterized among the MCRPE isolates revealed that MDR was a common phenotype in this study. *E. coli* resistance to beta-lactam and the tetracycline antibiotic groups was very common in Thailand, and aminoglycoside and fluoroquinolone resistance was found to be varied in farm management such as using antibiotic for prophylactic or treatment purposes (21). The MDR traits among *mcr-1*-positive *E. coli* have been reported frequently in pigs due to the usage of antibiotics in the production cycle (34). Interestingly, ESBLs were found at a high prevalence among the MCRPE isolates of this study, which might due to co-selection under selective pressure (33). Moreover, *E. coli* plasmids that harbor co-localization of *mcr-1* and *bla*_CTX−M_ genes and/or *mcr-1* and *bla*_NDM−5_ genes have been reported previously (35). Genomic characterization should be performed to resolve the reason for this apparent correlation.

The presence of the *Ltb, Stb, StaP, Stx2e*, and *K88* virulence genes in MCRPE isolates indicated that they also had the potential to cause an infection. Thus, healthy pigs could be an important reservoir of colistin-resistant ETEC. Interestingly, one MCRPE isolate was found to be an ETEC–EHEC hybrid strain. *E. coli* with highly virulent hybrid pathotype strains had been reported previously both in animals and human diarrhea patients (36). Since many of the virulence genes of *E. coli* are carried on mobile genetic elements, the genetic combination of these MGE resulted in the emergence of STEC/ETEC hybrid strains in multiple events (37). The recent finding of a clone of sequence type (ST) 95 showing extreme drug resistance with a high virulence potential underscores the need to monitor new and emerging trends in antibiotic resistance development in this important global lineage (38). On the other hand, aminoglycoside- and fluoroquinolone*-*resistant *E. coli* seemed to have a lower probability to act as an ETEC pathotype in this study. Pathogenic *E. coli* tends to be more susceptible to many antimicrobials (39). However, the mechanism is still not elucidated and clonal typing should be included for a more convincing analysis.

In conclusion, a low carriage rate of *mcr-1* and *mcr-3* co-positive *E. coli* was detected in large-scale contract pig farms in Thailand. The MCRPE isolates showed MDR *E. coli* and most of the isolates contained virulence genes representing an ETEC pathotype. These data provide an insight into the occurrence of colistin resistance among *E. coli* in healthy pig carriages and their characteristics, in terms of virulence genes and antibiograms. However, genomic characterization of *mcr* genes found in Thailand is required for further study.

## Data Availability Statement

The raw data supporting the conclusions of this article will be made available by the authors, without undue reservation.

## Ethics Statement

Ethical review and approval was not required for the animal study because all fecal samples were submitted from veterinarians in pig industrial field to the veterinary diagnostic laboratory as the annual surveillance. We asked the permission to use these sort of samples which did not directly collect the feces by our team. However, the biohazard execution control was approved by the Institutional Biosafety Committee of the Faculty of Veterinary Science, Chulalongkorn University (IBC 1731021).

## Author Contributions

All authors listed have made a substantial, direct and intellectual contribution to the work, and approved it for publication.

## Conflict of Interest

The authors declare that the research was conducted in the absence of any commercial or financial relationships that could be construed as a potential conflict of interest.
