# Supplementary material for: Multidrug Resistance and Virulence Factors of Escherichia coli Harboring Plasmid-Mediated Colistin Resistance: mcr-1 and mcr-3 Genes in Contracted Pig Farms in Thailand
Source: Front Vet Sci. 2020 Nov 10;7:582899. doi: 10.3389/fvets.2020.582899 (PMC7683614; doi:10.3389/fvets.2020.582899)
Supplement: Supplementary file 1 [file Data_Sheet_1.PDF]

Supplementary table 1. The list of primer sets used in this study for PCR detections

Gene Target	Primer	DNA sequence 5'→3'	Amplicon size(bp)	Refere-nces
mcr-1	mcr1_320bp_fw	AGTCCGTTTGTTCTTGTGGC	320	14
	mcr1_320bp_rev	AGATCCTTGGTCTCGGCTTG		
mcr-2	mcr2_700bp_fw	CAAGTGTGTTGGTCGCAGTT	715	
	mcr2_700bp_rev	TCTAGCCCGACAAGCATACC		
mcr-3	mcr3_900bp_fw	AAATAAAAATTGTTCCGCTTATG	929	
	mcr3_900bp_rev	AATGGAGATCCCCGTTTTT		
mcr-4	mcr4_1100bp_fw	TCACTTTCATCACTGCGTTG	1116	
	mcr4_1100bp_rev	TTGGTCCATGACTACCAATG		
mcr-5	mcr5_fw	ATGCGGTTGTCTGCATTTATC	1644	
	mcr5_rev	TCATTGTGGTTGTCCTTTTCTG		
chuA	chuA.1b	ATGGTACCGGACGAACCAAC	288	20
	chuA.2	TGCCGCCAGTACCAAAGACA		40
yjaA	yjaA.1b	CAAACGTGAAGTGTCAGGAG	211	20
	yjaA.2b	AATGCGTTCCTCAACCTGTG		
TspE4.C2	TspE4C2.1b	CACTATTCGTAAGGTCATCC	152	20
	TspE4C2.2b	AGTTTATCGCTGCGGGTCGC		
arpA	AceK.f	AACGCTATTCGCCAGCTTGC	400	20
	ArpA1.r	TCTCCCCATACCGTACGCTA		41
arpA	ArpAgpE.f	GATTCCATCTTGTCAAAATATGCC	301	42
	ArpAgpE.r	GAAAAGAAAAAGAATTCCCAAGAG		
trpA	trpAgpC.1	AGTTTTATGCCCAGTGCGAG	219	42
	trpAgpC.2	TCTGCGCCGGTCACGCCC		
trpA (internal control)	trpBA.f	CGGCGATAAAGACATCTTCAC	489	41
	trpBA.r	GCAACGCGGCCTGGCGGAAG		
parA-parB	HI1 FW	GGAGCGATGGATTACTTCAGTAC	471	22
	HI1 RV	TGCCGTTTCACCTCGTGAGTA		
iterons	HI2 FW	TTTCTCCTGAGTCACCTGTTAACAC	644	
	HI2 RV	GGCTCACTACCGTTGTCATCCT		

RNAI	I1 FW	CGAAAGCCGGACGGCAGAA	139	22
	I1 RV	TCGTCGTTCCGCCAAGTTCGT		
ori γ	X FW	AACCTTAGAGGCTATTTAAGTTGCTGAT	376	
	X RV	TGAGAGTCAATTTTTATCTCATGTTTTAGC		
repA,B,C	L/M FW	GGATGAAAACTATCAGCATCTGAAG	785	
	L/M RV	CTGCAGGGGCGATTCTTTAGG		
repA	*N FW*	GTCTAACGAGCTTACCGAAG	559	
	N RV	GTTTCAACTCTGCCAAGTTC		
iterons	*FIA FW*	CCATGCTGGTTCTAGAGAAGGTG	462	
	FIA RV	GTATATCCTTACTGGCTTCCGCAG		
repA	FIB FW	GGAGTTCTGACACACGATTTTCTG	702	
	FIB RV	CTCCCGTCGCTTCAGGGCATT		
repA	W FW	CCTAAGAACAACAAAGCCCCCG	242	
	W RV	GGTGCGCGGCATAGAACCGT		
repA	Y FW	AATTCAAACAACACTGTGCAGCCTG	765	
	Y RV	GCGAGAATGGACGATTACAAAACTTT		
iterons	*P FW*	CTATGGCCCTGCAAACGCGCCAGAAA	534	
	P RV	TCACGCGCCAGGGCGCAGCC		
repA2	FIC FW	GTGAACTGGCAGATGAGGAAGG	262	
	FIC RV	TTCTCCTCGTCGCCAAACTAGAT		
repA	A/C FW	GAGAACCAAAGACAAAGACCTGGA	465	
	A/C RV	ACGACAAACCTGAATTGCCTCCTT		
repA	T FW	TTGGCCTGTTTGTGCCTAAACCAT	750	
	T RV	CGTTGATTACACTTAGCTTTGGAC		
repA	FIIS FW	CTGTCGTAAGCTGATGGC-	270	
	FIIS RV	CTCTGCCACAAACTTCAGC		
RNAI/repA	FrepB FW	TGATCGTTTAAGGAATTTTG	270	
	FrepB RV	GAAGATCAGTCACACCATCC		
RNAI	K/B FW	GCGGTCCGGAAAGCCAGAAAAC	160	
	K RV	TCTTTCACGAGCCCGCCAAA		
RNAI	B/O RV	TCTGCGTTCCGCCAAGTTCGA	159	

STb (*estB*)	STb FW	TGCCTATGCATCTACACAAT	113	23
	STb RV	CTCCAGCAGTACCATCTCTA		
STaP (*estA*)	STaP FW	CAACTGAATCACTTGACTCTT	158	
	STaP RV	TTAATAACATCCAGCACAGG		
K99 (*fanA*)	K99 FW	AATACTTGTTCAGGGAGAAA	230	
	K99 RV	AACTTTGTGGTTAACTTCCT		
LTb (*eltB*)	LTb FW	GGCGTTACTATCCTCTCTAT	272	
	LTb RV	TGGTCTCGGTCAGATATGT		
F18 (*fedA*)	F18 FW	TGGTAACGTATCAGCAACTA	313	
	F18 RV	ACTTACAGTGCTATTCGACG		
987P (*fasA*)	987P FW	AAGTTACTGCCAGTCTATGC	409	
	987P RV	GTAACTCCACCGTTTGTATC		
K88 (*faeG*)	K88 FW	GTTGGTACAGGTCTTAATGG	499	
	K88 RV	GAATCTGTCCGAGAATATCA		
F41 (*fedA* subunit)	F41 FW	AGTATCTGGTTCAGTGATGG	612	
	F41 RV	CCACTATAAGAGGTTGAAGC		
Stx2e (A subunit)	Stx2e FW	AATAGTATACGGACAGCGAT	733	
	Stx2e RV	TCTGACATTCTGGTTGACTC		

References (supplementary table)

40. Clermont O, Bonacorsi S, Bingen E. Rapid and simple determination of the Escherichia coli phylogenetic group. *Appl Environ Microbiol* (2000) 66(10):4555-8. Epub 2000/09/30. doi: 10.1128/aem.66.10.4555-4558.2000. PubMed PMID: 11010916; PubMed Central PMCID: PMCPMC92342.

41. Clermont O, Bonacorsi S, Bingen E. Characterization of an anonymous molecular marker strongly linked to Escherichia coli strains causing neonatal meningitis. *J Clin Microbiol* (2004) 42(4):1770-2. Epub 2004/04/09. doi: 10.1128/jcm.42.4.1770-1772.2004. PubMed PMID: 15071045; PubMed Central PMCID: PMCPMC387582.

42. Lescat M, Clermont O, Woerther PL, Glodt J, Dion S, Skurnik D, et al. Commensal Escherichia coli strains in Guiana reveal a high genetic diversity with host-dependant population structure. *Environ Microbiol Rep* (2013) 5(1):49-57. Epub 2013/06/13. doi: 10.1111/j.1758-2229.2012.00374.x. PubMed PMID: 23757130.

Supplementary Table 2. Control strains used in antimicrobial susceptibility testing and PCR in this study

Positive controls	Methods	References
E. coli CUP13	*mcr-1-5*	15
E. fergusonii CUVET427	Phylogroup	21
P27 phage	Virulence genes	24
pEWD299	Virulence genes	24
pDAS100	Virulence genes	24
pDAS101	Virulence genes	24
E. coli PCU12_1	Replicon typing	21
E. coli PCU13_1	Replicon typing	21
E. coli CUVET32_1	Replicon typing	21
